# *“A life-changing experience and the beginning of a lifelong commitment”*: experiences and impact of Global Health Community Service-Learning in undergraduate dental curriculum in Canada

**DOI:** 10.1186/s12903-024-04539-5

**Published:** 2024-07-15

**Authors:** Abbas Jessani, Alexia Athanasakos, Samantha Kachwinya

**Affiliations:** 1 Department of Dentistry, Schulich School of Medicine and Dentistry, Western University, London, ON Canada; 2 Department of Epidemiology and Biostatistics, Schulich School of Medicine and Dentistry, Western University, London, ON Canada; 3 School of Dentistry, College of Health Sciences, Makerere University, Kampala, Uganda

**Keywords:** Global health, Community Service-Leaning; Reflective journals, Dental curriculum

## Abstract

**Background:**

Global Health Community Service-Learning (GHCSL) can have a profound professional and personal impact on learners. This pedagogy provides understanding of unfamiliar environments and challenges learners to step out of their comfort zones, adapt to new cultures, and navigate unique situations. Yet, there are relatively few studies exploring the experiences of learners participating in community service-learning placements in global regions as part of undergraduate dental curriculum. This study aimed to explore the experiences and impact of the GHCSL program in East Africa among undergraduate dental learners at the Schulich School of Dentistry.

**Methods:**

Eight undergraduate dental learners were enrolled in GHCSL pilot placements. Placement agreements were established with Makerere University in Kampala, Uganda, and the University of Rwanda in Kigali, Rwanda for the summer of 2022. Stakeholders from both institutions were engaged in the development and implementation of these placements. Learners were required to engage in weekly reflection through a ‘storytelling and incident-based narrative’ while carrying out their placement. A qualitative study design was employed, and an inductive interpretive approach was utilized to thematically analyze the learners’ reflective essays.

**Results:**

Five major themes emerged from the learners’ reflective essays: (1) experiential clinical learning; (2) cultural humility and social awareness; (3) awareness of contrasting healthcare systems; (4) commitment to service; and (5) personal and professional growth. Most learners reflected on their engagement with diverse communities, being exposed to unique patient cases, and witnessing the adaptability exuded in resource-constrained environments. These experiences presented the learners with an opportunity to develop cultural humility and gain a newfound motivation to mitigate global oral health disparities in populations beyond that of their local communities. Learners also reflected on enhanced social awareness experiences and the awareness of contrasting healthcare systems in Canada and their placements, which encouraged the development of empathy, communication, and compassion skills, as well as an understanding of the disproportionate burden of conditions in low-resource settings.

**Conclusion:**

The reflective essays concluded that the GHCSL placements had a positive impact on the learners, encouraging many to develop a heightened desire for lifelong learning to address oral health disparities within a global context.

## Introduction

The burden of oral diseases still prevails especially among socially disadvantaged and vulnerable people, regardless of their geographical location [1, 2]. Therefore, a shift from the treatment-dominated model of dentistry to a sustainable preventative approach, focusing on population-wide effects that address persistent inequalities, is necessary [1]. The current dental curriculum model inadequately prepares oral health providers to address population-wide oral health needs and deliver evidence-based care, primarily due to the teaching of reactive and surgical approaches to care and limited experiential learning opportunities in global settings [1]. To effectively tackle the global burden of oral diseases, a radically different approach that integrates experiential learning and Global Health Community Service-Learning (GHCSL) within the wider structure of dental curriculum must be advocated for [3].

GHCSL is an experiential pedagogy that combines academic instruction with the provision of mutually beneficial services that meet the needs and goals of a global community through outreach initiatives and partnerships [4]. Meaningful GHCSL fosters a sense of social responsibility, encourages lifelong community engagement, and promotes the development of empathetic and ethical values, as well as critical thinking and problem-solving skills [3, 5]. Learners can further embrace the notion of global citizenship as they prepare to engage with diverse cultures and global experiences not limited to their local institution [6].

Although GHCSL has been reported to have academic benefits for learners, the design and implementation of such programs must be guided by principles of mutuality, community engagement, and capacity building through established partnerships, while sustainability addressing the needs of the local community [6, 7]. According to Melby et al. [8], short-term experiences in global health (STEGH), such as the one’s offered by the GHCSL program, must be designed with the following components: (1) skills building in cross-cultural effectiveness and cultural humility, (2) bidirectional participatory relationships, (3) sustainable local capacity building and health systems strengthening, and (4) community-led efforts focused on sustainable development. Broad categories to aid in the integrations of such components include: pre-departure training focused on cultural humility, defined goals with the engagement of local stakeholders that focus on community development rather than solely learner skills, and expectation management that focuses on equity, the prioritization of the local community, and the achievement of sustainable outcomes on needs identified by the local community such as unmet oral health needs [7–10].

Untreated oral health conditions such as tooth decay, periodontal disease, and dental caries, are major public health problems in East Africa [11–13]. A community-based oral health survey in Uganda reported that just over half of adults in Ugandan communities had at least one oral health concern, and of these, only about one-third received appropriate treatment [12]. Similarly, the Rwanda National Oral Health Survey in 2018 found that the oral health status of Rwandan communities was poor, with 70 percent of people not utilizing oral health services [13]. Underlying factors such as poor oral health-related attitude and knowledge are highly reported barriers for the utilization of oral health services as the adverse notion that dental clinics are to be visited once symptoms such as dental pain or discomfort arise is still considered a ‘norm’ in East Africa [12–15]. Cost of oral health care is an additional barrier to accessing oral health services [12]. For families residing in low- and middle-income countries like Uganda and Rwanda, the cost of oral health care can significantly impact their household income, with many at greater risk of falling below their country’s poverty line once oral health care costs are subtracted from their household income [16]. As such, the utilization of oral health services is often neglected on the basis of low-income [12]. Those of low-income also often reside in rural regions, where access to oral health services remains a challenge in both Uganda and Rwanda due to urban bias, one’s social determinants of health, transportation-related barriers, and time constraints [17, 18]. For instance, in Uganda, among the 40 districts containing over 90 percent of the rural population, 55 percent had one dental clinic, and 20 percent had none [19]. A gap in the literature remains regarding the geographic availability of dental clinics in Rwanda; however, in general, across low- and middle-income countries, oral health service availability reduces as the proportion of rural and low-income populations increases [19].

Across Uganda and Rwanda, the practice of seeking routine oral examinations as a preventive measure for common oral diseases is not widely known or implemented [12–15]. Therefore, there is a need for oral health service-learning programs that could help serve the unmet oral health needs of local communities and provide educational training, with a focus on cross cultural exchange, to undergraduate dental learners by meaningfully reflecting on social determinants of health.

Reflective narratives are considered an integral component of GHCSL as they enable learners to gain additional value from CSL experiences and positively impact the attitudes of learners regarding services and the community itself [20, 21]. Guided reflective journals have been widely used within undergraduate professional curriculums, including medical, dental, and other health sciences programs [22]. Through reflective thinking, learners are provided the opportunity to interpret and construct meaning to experiences; critically assess their assumptions, biases, and cultural perspectives; and gain knowledge—enhancing not only their ability to become more ‘self-aware’ of their learning but their development of cultural humility and personal growth [20, 23, 24]. For example, Kolb’s [25] experiential learning theory, which was integrated into the GHCSL program’s reflective assignment component, describes reflective practice as a cycle of experiences, reflection, conceptualization, and experimentation. This metacognition exercise enable learners to “fit together” the thoughts, feelings, and values of their experience into an overall “picture” that showcases the evolution of experience into knowledgeable skills [21, 25, 26].

Given the lack of studies investigating the experiences of undergraduate dental learners participating in GHCSL placements, this study aimed to explore the experiences and impact of GHCSL within the undergraduate dental curriculum by analyzing the guided reflective essays of undergraduate dental learners.

## Methods

### Guiding principles and framework

The GHCSL program is an experiential learning component of the Community Service-Learning (CSL) program at the Schulich School of Dentistry, Western University. The CSL program was developed and piloted in the Winter term of 2021. The main objectives of the CSL program are as follows: 1) to address the high unmet oral health treatment needs of equity-seeking community members such as people living with human immunodeficiency virus (HIV); high-risk youth facing housing and addiction crises; refugees and immigrants; Indigenous peoples; and Two-Spirit, lesbian, gay, bisexual, transgender, queer (or questioning), and other sexual orientations and gender identities (2SLGBTQ +) people, and 2) to develop experiential community-based training opportunities for undergraduate dental learners with a focus on person-centred and trauma-informed care. During the academic year, third year (D3) and fourth year (D4) learners are placed in various local community organizations throughout Southwestern Ontario to address the unmet oral health needs of local community members.

Yoder’s (2006) [27] framework for service-learning in dental curriculum was adapted to develop the CSL and subsequent GHSCL program. The framework consists of ten components that collectively offer a structured approach for the planning, implementation, and evaluation of service-learning in dental curriculum [27]. The use of this framework allowed learners to differentiate between service-learning and other types of local and global community engagement, enabling a more precise understanding of the characteristics of each global placement [27]. Community engagement was a key component in the development of the GHCSL program through collaborating with stakeholders from Makerere University in Kampala, Uganda and the University of Rwanda in Kigali, Rwanda. These stakeholders were actively engaged in the development of the GHCSL program and its objectives, as well the service-learning activities, based on the needs of the populations to be served by the learners.

Lave and Wenger’s (1991) [28] theory of situated learning served as one of the guiding principles for the development of the CSL and subsequently the GHCSL component. This theory offers a perspective on learning that steers away from individual-focused, cogitative, and behaviourist views that historically dominated educational systems [29]. The characteristics of learning, as emphasised by the theory, are considered to be fundamentally social; embedded in everyday activity, context, and culture; unintentional rather than deliberate; and progressive with respect to learners’ participation [28, 29]. The theory emphasizes relationships and interactions between *newcomers* and *more knowledgeable others* as a means to showcase the learning of knowledgeable skills as a dynamic process of guidance, support, and co-development [28, 29]. With this analytical viewpoint in mind, the CSL and GHCSL program was designed to encourage the immersion of undergraduate dental learners in a “community of practice,” providing them the opportunity to gradually develop awareness of behaviours characteristic to the community to address their unmet oral health needs [5, 30].

Learners were also familiarized on Kolb’s (1984) [25] experiential learning theory prior to their placement experiences as part of the pre-departure training. The theory emphasizes effective reflective learning as a four-stage learning cycle: of (1) having a new experience followed by (2) reflection on that experience which leads to (3) the formation of new ideas and the adaption of prior knowledge which are then (4) applied to real-world situations. Such cycle acted as a systematic structure to the learners as they engage in weekly reflection through a ‘storytelling and incident-based narrative.’

### Implementation of the GHCSL program

In the academic year of 2022–23, the Schulich School of Dentistry, Western University, piloted two elective courses, D5401 and D5010, with CSL placements in Southwestern Ontario and GHCSL placements in East Africa. The main objectives of the GHCSL program are as follows: 1) to learn about and address the unmet oral health needs of those in low-resource global settings and 2) to provide undergraduate dental learners with experiential learning opportunities, emphasizing community-engaged learning, social accountability, and person-centred care. These objectives were developed based on the strategic priorities of the Schulich School of Dentistry in conjunction with the stakeholders from Makerere University and the University of Rwanda. The GHCSL program director (AJ) and co-author (SK) had director roles relative to the GHCSL placements.

Placement agreements were established with Makerere University and the University of Rwanda for the summer of 2022. Both sites were selected based on their long-standing partnerships with Western University and their alignment with the mission, vision, and values outlined within the Schulich School of Medicine and Dentistry’s 2021–2026 Strategic Plan. All institutions involved prioritized community engagement, leading to the collaborative and mutually agreed-upon development of the GHCSL program’s objectives. This approach ensured that needs and priorities of the local communities were adequately considered, and unilateral “aid” was avoided [8]. The Strategic Plan also emphasized the fostering of strong partnerships as a critical aspect to the successful expansion of CSL opportunities and embraced the notion that learners must continually advance and evolve their knowledge and skills to identify and address the priority health needs of people within and beyond the institutions borders [31].

### Selection process

Rigorous criteria were established for learners’ recruitment and selection. The criteria in this process included critical thinking, demonstration of cultural humility and professionalism during the local CSL placement, and their ability to adapt in response to identified needs in the host community [32]. During the selection processes, an interdisciplinary committee consisting of staff from The African Institute at Western University, faculty members from the Schulich School of Dentistry and host institutions, and administrators evaluated the learner’s blinded applications with didactic grades, clinical performance, a letter of motivation, and letter of references (Appendix 1). Competitive candidates were selected, and a waiting list was created. The number of learners selected for participation reflected the holding capacity of the placement sites.

### Learner preparation, training, and debrief

Pre-departure training, offered by Western’s International Learning, was a requirement for all learners participating in the GHCSL program. This training focused on, health and wellness, cyber security, intercultural engagement, safety abroad, travel logistics, and critical and ethical global engagement. To successfully complete the training, learners must have achieved at least 80 percent in the final quiz within two attempts and upload the electronic certificate of completion to their Travel Registry entry through Western's International Learning portal. Pre-departure semi-structured virtual seminars were also held with local mentors from the host institutions to outline placement-specific protocols and responsibilities; familiarize learners on local cultural beliefs, health practices, cross-cultural communication methods, and the role of social determinants of health in oral health access; and co-create individualized learning objectives reflective of each learners’ educational goals needs. Local mentors, were oral health providers who worked and received training at either Makerere University or the University of Rwanda. Failure to successfully complete the pre-departure training and attend all pre-departure sessions would result in the learner being held back, as this would be considered a sign that they do not demonstrate the required competencies. Given the language and power differentials between learners and the local communities, the pre-departure training taught an “explanatory models” approach, along with cultural humility, providing a foundation of understanding and preparation for the learner that ensures the value of knowledge of local stakeholders over preconceptions [8]. Without this understanding and preparation, STEGH would be more likely to cause harm and less likely to contribute meaningfully to the local community [32]. Post-return debriefing sessions were held with the GHCSL program director and the director of Western International as an evaluation and feedback tool to discuss the learner’ experiences, strengths of the program, and suggestion to support continuous improvements [32]. Similar feedback was obtained from the local mentors and stakeholders to ensure that expectations were met.

### GHCSL program streams

The GHCSL program comprised two streams: pre-clinical and clinical. The stream in which a learner was placed was dependent on their year of study—D3 learners completed the pre-clinical stream, and D4 learners completed the clinical stream. Learners enrolled in the pre-clinical stream successfully completed two years of pre-clinical training at the Schulich School of Dentistry and were permitted to observe and assist in procedures under the supervision of local mentors. Learners enrolled in the clinical stream successfully completed a full year of clinical training at the Schulich School of Dentistry and were permitted to, in addition to observing and assisting, provide clinical care to those in low-resource settings under the supervision of local mentors. Each learner’s ability and degree of independence was accessed by their local mentor once at their placement site to avoid the harmful assumption that the level of independence in novel low-resource settings would mirror that of learner’s local institute [8]. Language and cultural differences between learners and host communities, as well as novel standards of care and treatment protocols, suggest that it is more appropriate for learners to have less independence and scope of practice to avoid the degradation of professional and ethical standards [8].

Learners were also paired with local undergraduate dental learners from their respective placement sites to aid the learner’s ability to integrate and engage with community members; gain insight into the local standards of care and cultural beliefs; and learn key words and phrases in the local language to show respect for the culture of the local community, as English proficiency was not widespread in Uganda and Rwanda [8]. The GHCSL program director joined the learners for the first week of their placements to immerse in the experience for quality improvement purposes.

### Reflective journals and data collection

A total of eight undergraduate dental learners were enrolled in the pilot GHCSL program—six completed their placement at Makerere University for a duration of three weeks, and two at the University of Rwanda for three and a half weeks. Of these, four were D3 learners and four were D4 learners. The placements consisted of experiential learning and didactic components. In concomitance with the experiential learning component, the learners were required to engage in weekly reflection through a ‘storytelling and incident-based narrative’ while carrying out their GHCSL placement (Appendix 2). At the end of their placement, each learner compiled their weekly journals into one reflective essay for submission to Western University’s online learning management system. The reflections were not graded but assessed pass or fail. To pass, learners had to favour reflective thinking on the individualized and personal stories of the community members screened (i.e., the challenges and social determinants of health and access to dental care within the community) and the impact these experiences may have on their roles as future oral health providers. Feedback was provided by the GHCSL program director on their ability to conceptualize, critique, and reflect on their experiences, but edits were not permitted once reflective essays were submitted for assessment [21, 25]. Learners were encouraged to discuss concerns or comments made with the GHCSL program director.

### Data analysis

Ethical approval for this study was obtained from the Western University Behavioural Ethics Board. All eight learners (*N* = 8) consented to data anlaysis of their reflective journals. To maintain confidentiality, each essay was de-identified with no identifiable information such as name, student number, etc. made visible. De-identified essays were then stored in a password protected OneDrive folder, accessible only to the research team. The journals were transported and stored in the NVivo 14 software program for data analysis.

The content of each reflective essay was thematically analyzed through the utilization of an inductive interpretive approach. The initial step entailed researchers (AJ and AA) independently reading each reflective essay to familiarize themselves with the content and recognize fundamental thoughts [33, 34]. Each essay was line-by-line coded using the NVivo 14 software program [35, 36]. AJ and AA independently developed as many codes as necessary to describe all aspects of the content, then met to discuss their analysis [33, 34]. Next, selective coding took place and similar codes were combined to generate major themes related to the aim of the study (Table 1) [35, 36]. Excerpts from the essays were then extracted to illustrate these particular themes and associated ideas that represented most effectively the range of experiences, opinions, meanings, and feelings described by the learners [37, 38].

**Table 1:** Thematic analysis of reflective essays

Theme 1 Experiential clinical learning	
Codes	Technique adaption, clinical learning experiences, dental procedure disparities, limited resources, resource adaption, HIV prevalence awareness, familiar clinical protocols, heterogeneity of beauty standards, stigmatization of HIV
Theme 2 Cultural humility and social awareness	
Codes	Bonding through shared experiences, common values, poverty, low-income, barriers to care, patient communication, language barriers, importance of consistent communication, importance of interprofessional collaboration, gain insight into Ugandan culture, learning the local language, deeper connections, cultural immersion, commitment to common goal, knowledge sharing, cultural exploration, inclusive atmosphere
Theme 3 Awareness of contrasting healthcare systems	
Codes	Funding disparities, low education, patient charting differences, casualties ward, turnover rate, healthcare based on ability to pay, prioritize early intervention, disparities in healthcare access, disorganization within medical system, resourcefulness, resource constraints, country-specific challenges, financial considerations, health-seeking behaviour, government policies
Theme 4 Commitment to service	
Codes	Passion for advocacy, commitment to improving healthcare access, word towards addressing the social determinants of health, commitment to advocating for improved healthcare access, deep passion to advocate for a better healthcare system, privilege, newfound direction, clarity
Theme 5 Personal and professional growth	
Codes	Empathy building, communication building, broadened understanding, prioritization of patient comfort, continuous improvement, patient monitoring, assessment of patient needs, perspective shifts, clinical skills, compassion building

**Fig. 1:**
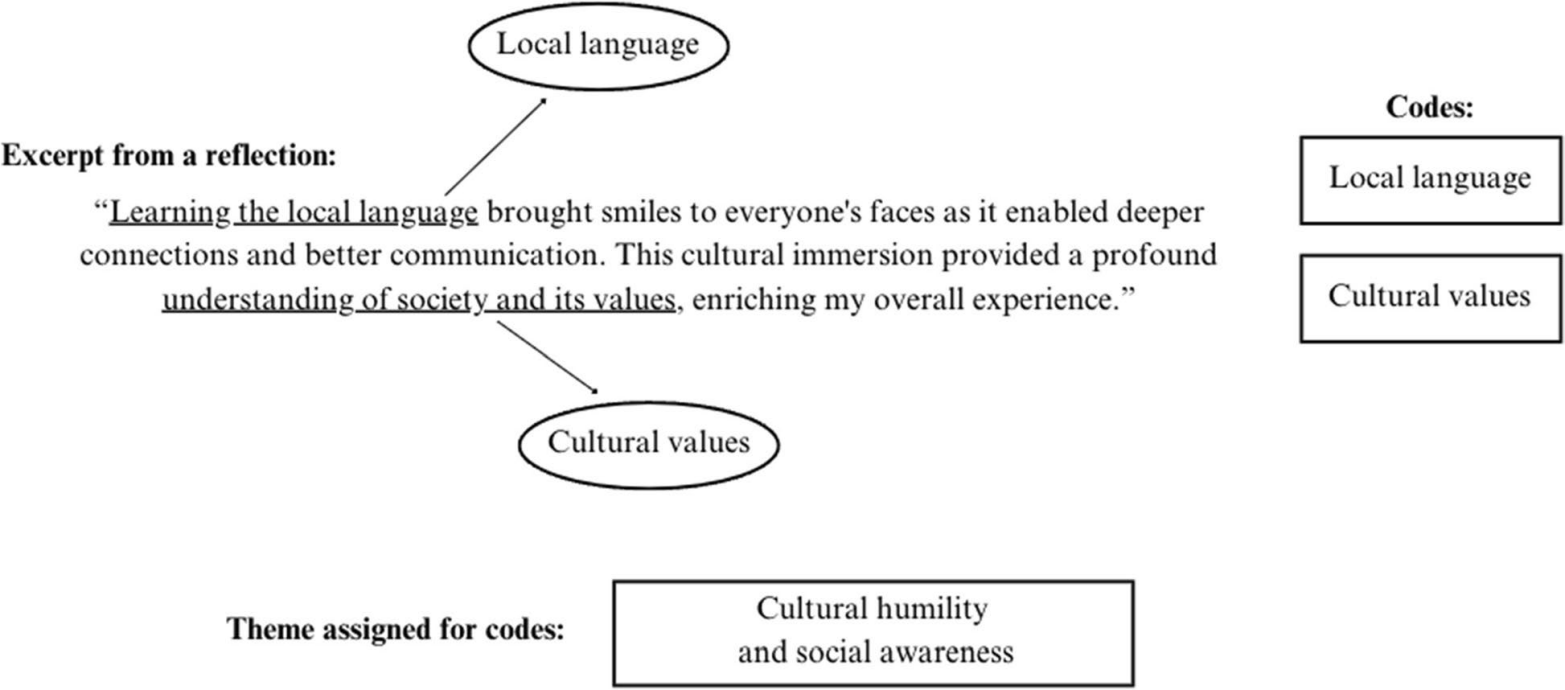
Example of a thematic map developed for the theme “cultural humility and social awareness.”

To ensure rigor and accurate interpretation, AJ and AA attended to the thoroughness, coherence, and comprehensiveness of the emerging themes from each reflective essay as major themes were identified [38]. Constant comparison allowed for a thorough analysis in which codes assigned to excerpts of the reflections were gathered together in the format of a map (Fig. 1). Rather than quantifying the number of experiences or perspectives identified, the goal was to provide examples of the multiple viewpoints described by the learners to provide the reader with a range and depth of experiences, challenges, and emotions that the learners reported [38].

## Results

The major themes that emerged from the reflective journals were: (1) experiential clinical learning; (2) cultural humility and social awareness; (3) awareness of contrasting healthcare systems; (4) commitment to service; and (5) personal and professional growth. These themes intersect and/or compile the experiences of the learners while carrying out their GHCSL placements (Schwab et al., 2024).

### Experiential clinical learning

The multifaceted nature of the learners’ clinical experiences was highlighted throughout various reflective narrations, with emphasis placed on the intersection of clinical practice, cultural safety, and public health advocacy in addressing global oral health disparities. For example, one learner noted that their placement provided *a great experience in understanding the oral diseases and pathologies that present within the population*, which helped them *to understand that patient management works very similarly within global setting.*

Incidences where the learners were exposed to cases seldom encountered within the learner’s local institution's clinic were also mentioned. One learner said,

*“We got to see a lot of cases that we usually don’t see at the Schulich clinic and have only read about within our lecture notes or in our textbooks. It really made me understand that it’s important to understand the management of such cases for when they do actually present themselves.”*

The learners appreciated the opportunity to compare and contrast clinical protocols between the learner’s local institution and their placement site, noting similarities in procedural aspects while acknowledging the nuances. During a learner’s periodontal rotation at Makerere University, they reflected on how *in depth the information gathering was on extra-oral features* compared to that of their local institution’s clinic. The learner went on the share that local mentors would use this information as indicators in *the general well-being of the patient and their oral care.* Other learners noted the influence of cultural practices on the heterogeneity of beauty standards, with one learner stating the following:

*“What really stood out to me was how many dentures I saw with a midline diastema. My clinic partner explained to me that a lot of the patients actually request to have a midline diastema in their dentures as diastemas are seen as a symbol of beauty in Rwanda culture.”*

The learners also gained first-hand insight into the experiences of people living with infectious diseases such as HIV infection. They observed instances of stigma and discrimination directly towards these patients, as shared by the following learner:

*“She was an HIV positive patient and present in pain and required multiple teeth to be extracted. She told us that she had been to many other local clinics but was denied the care due to the stigmatization of her condition.”*

Despite these challenges, the learners observed initiatives aimed at promoting research and advancing understanding of prevalent health issues, such as HIV infection. For example, one learner observed a community of HIV-positive people wanting to participate, in any way they could, towards the progression of research. It was the number of participants that the learner witnessed *that emphasized the prevalence of HIV in Uganda*, *[…] highlighting the importance of the research performed and the impact it could have.*

### Cultural humility and social awareness

The reflective essays indicted an understanding of cultural diversity and the building of foundational skills in cross-cultural effectiveness. When providing care to local community members, learners reflected on the importance of navigating diverse cultural contexts with *a high degree of sensitivity and adaptability*. The implementation of culturally sensitive healthcare practices was emphasized among learners. They reflected on the understanding of *local customs and communication styles* as integral components of *building trust and providing effective care.*

GHCSL provided the learners a heightened understanding of social determinants of health on oral health, with one learner sharing the following:

*“The consequence of living in poverty left [local community members] with limited resources to invest in education, healthcare, and, notably, dental care. This circumstance partly explains why many of the patients I encountered at the public hospital dental clinic presented with rampant dental caries and non-restorable teeth. The profound impact of poverty on their oral health and overall well-being has left an enduring impression on me.”*

The cultural and language differentials between the local communities and the learners led many on the role of observer and learner during clinical rotations, asking questions about the local culture, language, and health needs of the community from local learners and mentors to build their cultural humility. One learner shared the following:

*“[…] my clinic partner taught me a few Kinyarwanda [the national language of Rwanda] words that would be useful to me when we went into the clinic tomorrow. He wrote down a few words and said that he would test me tomorrow.”*

For another learner, social interactions with local mentors, learners, and community members allowed them to learn common phrases in *Luganda, the local language [of Uganda], and gain insights into Ugandan culture, particularly their family structures and social customs.* The learner went on the share that such doings *enabled deeper connections and better communication* skills. A sense of comfort and belonging was further reflected on by learners, with one learner sharing the following experience:

*“Perhaps the most profound aspect of the day was the genuine connections forged with the locals […] that transcended the boundaries of nationality. We become not just visitors but participants in a shared human experience. The lessons learned here, the friendships formed, and the challenges faced will undoubtedly stay with me long after the plane takes me back home.”*

Another learner said,

*“We landed mere hours ago, yet I already feel a deep sense of belonging. The people of this community, warm and resilient, have welcomed us with open arms.”*

### Awareness of contrasting healthcare systems

Learners compared and contrasted the healthcare systems of their home country of Canada and their placement site, sharing key distinctions in terms of the resource availability, efficiency, and severity of conditions presented, while also reflecting on the similar barriers faced by the patient populations in London (Ontario) and practicum sites in Uganda and Rwanda. One learner said,

*“Reflecting on the challenges and social determinants of health, it became evident that these disparities were, in some ways, similar to those in Canada. The global issue of access to dental care extended beyond borders. In both settings, there were underserved populations who lacked access to basic dental services due to financial constraints or geographical factors. […] These stark disparities in healthcare access and resources that I witnessed during my time in Uganda have […] served as a reminder that access to advanced dental care should not be a privilege but a basic right for all.*

One learner went on to note that *a striking difference lie[d] in the availability of support system* with many people having *no choice but to rely solely on themselves* while it was expressed that in London (Ontario), *organizations and programs are accessible to CSL patients, providing assistance when needed.*

The noted differences extended beyond barriers and support, with resource allocation in Canadian dental care settings contrasting sharply with the lack thereof in Uganda. Some learners noted that they became increasingly aware of the *severe shortages of personal protective equipment* during their placement. One learner said,

*“This experience was a strong reminder of the disparities in healthcare systems and the ethical dilemmas that arise when resources are scarce. This experience prompted me to consider the significance of adaptable and culturally sensitive healthcare practices, emphasizing the need for global collaboration to ensure that no patient, regardless of their location, is left to suffer due to inadequate access to care.”*

Despite these shortcomings in Uganda and Rwanda, learners highlighted the resourcefulness and dedication of local healthcare systems in delivering dental care to underserved populations, particularly when compared to Canada. One shared the following insight on the matter:

*“Although we have so much at our disposal in Canada, we still have populations who receive little to no care under the guise of lack of supplies and funding. Yet as I look around in Mengo clinic, where supplies are beyond limited, patients are having their oral health needs attended to. While compromising on health and safety measures is obviously not the answer, the resourcefulness I witnessed oversees is paradoxically proof that we are not doing enough in Canada towards serving the underserved.”*

Common trends in certain conditions were highlighted, such as *a very high prevalence of periodontal diseases within the population,* compared to that of Canada *as many [community members] are not educated on oral hygiene maintenance and rarely seek dental care*. A high prevalence of HIV was witnessed as well with one learner sharing that the *sheer number of HIV patients* encountered through their placement *emphasized the prevalence of HIV in Uganda*. Additionally, along with a high prevalence of HIV was a heightened degree of knowledge regarding the prevalent conditions witnessed within local healthcare systems. As expressed by one learner, the *clinician did not treat them [HIV-positive patients] any differently*—*there was no use of double gloves or stigmatization. *The learner went on to share that the patients were seen *beyond the diagnosis* and treated *with dignity and respect*, a sentiment that they feel is *still not achieved in Canada*.

### Commitment to service

GHCSL resulted in a greater appreciation of the health advocate, with learners reflecting on their motivation and dedication to mitigating global oral health disparities. A number of affirmations were expressed, including: 1) engaging *in further outreach initiatives,* 2) returning *to Uganda to provide better health and dental care for children,* 3) *advancing initiatives aimed at improving access to dental care for marginalized communities,* 4) pledging to *play a part in the ongoing narrative of empowerment and oral health in Uganda,* and 5) establishing *a free dental clinic in Uganda in the future.*

Furthermore, having been exposed to the unmet dental needs of the local community, one learner stated,

*“These experiences served as a powerful reminder of the tremendous disparities that exist on a global scale and highlighted the importance of addressing poverty and its multifaceted consequences. It reinforced my commitment to advancing initiatives aimed at improving access to dental care for marginalized communities.”*

Another learner said,

*“The experiences in Uganda have also ignited a passion for advocacy and a commitment to improving healthcare access and resources, not only locally but on a global scale. I'm driven to work towards addressing the social determinants of health and advocating for equitable healthcare for all, regardless of geographical location or financial means.”*

### Personal and professional growth

The learners felt that the placements facilitated a deeper self-awareness, both *personally* and *professionally*. Some learners reflected on the influence of social determinants of health in the context of global health. The learners reflected on their ability to adapt to the challenges of working in resource-limited environments while gaining appreciation for the practices demonstrated by their peers and mentors. One learner said,

*“This dental outreach experience deepened my understanding of the intricate interplay between access to care and socioeconomic factors. It broadened my understanding of global healthcare disparities and invoked my passion to addressing these issues. It served as a powerful reminder of the importance of accessible, equitable, and compassionate healthcare for all, regardless of socioeconomic status or geographic location. This transformative journey will continue to shape my perspective and actions in the field of dentistry.”*

Some learners also commented that their experience encouraged them to gain a deeper insight into providing dental care within resource-constrained environments*.* One learner expanded on the influence of adaption, sharing that:

*“This experience has also made me more adaptable(…). It makes me feel more comfortable thinking about partaking in future outreach efforts locally and globally, to help eliminate some barriers to care.”*

The learners also referred to difficulties in communication, notably when confronted with the realization that language barriers could complicate interactions with their patient population. It was later noted, often during the final week of their placements, that the learners came to appreciate through their experience *that effective communication is not solely dependent on a language*--; it also relies on *empathy and a shared commitment to a common goal*. Other learners went on to reflect that decisions cannot be made in isolation, especially when interacting with numerous healthcare providers to provide patient care as *it can significantly compromise the quality of patient care, increase the risk of medical errors and complications, and result in patient distress*. One learner then remarked that observing the adverse consequences stemming from inadequate communication among various healthcare providers during their placement led them to fully *appreciate why we emphasized the importance of effective interprofessional collaboration to provide individualized, high-standard care.* The importance of communication, specifically with patients, was also referred to by the learners:

*“As dental professionals, we have a unique privilege to get to know our patients and participate in the continuity of care by engaging in conversation. Yet, this is often forgotten. Going forward, I will make a habit of checking in on my patients and referring them to the appropriate health care providers and resources if needed.”*

## Discussion

Current literature confirms the importance of CSL and the benefits of experiential clinical learning curriculum on the attitudes and knowledge of learners participating in such outreach initiatives; however, there remains a scarcity of literature related to the integration of CSL within a global context into undergraduate dental curriculum. The study described here is novel in its exploration of the experiences and impact of GHCSL among undergraduate dental learners through the utilization of reflective essays. We found five overarching themes that intersect and/or compile the experiences of the learners while carrying out their GHCSL placements—these themes are to represent the preliminary findings of this pilot program at the Schulich School of Dentistry, Western University. Similar to other studies, the results of this study demonstrated positive findings and skills building in cultural humility, understanding of social determinants of health, and positive attitudes towards service-learning among dental learners [39, 40]. The benefits of GHCSL for local communities were also optimized through the fostering of bidirectional participatory relationships and local capacity building. Through the engagement with local communities, the GHCSL program director developed continuing professional development seminars and community-based participatory research projects reflective of the local needs identified by the local communities and institutions.

Reflections revealed that these positive findings were influenced by learners’ ability to compare and contrast their education in the context of global health unique to their home community and institution. Studies have revealed that experiential learning, derived from “real-life” clinical experiences in low-resource settings, can substantially compliment the development of clinical competencies and cultural humility [22, 41, 42]. However, to make such an assertion, learners from high-income countries must receive adequate training to develop an understanding of socioeconomic barriers, cultural beliefs, and emotional needs, as well as ask questions centred around the historical causes of the health inequities pertinent to the local context [9]. Truong et al. [43] suggested that experiences in global health may foster a broader perspective on global oral health disparities among learners, encouraging them to advocate for oral health equity and gain a desire to seek solutions that address inequities.

Emphasis has been placed by the learners on their emerging understanding of the local culture, language, and health needs of the community as they took a step back in their scope of independent activities to learn and observe from local mentors, learners, and community members. Studies have recognized CSL as a pedagogy for providing learners the opportunity to build skills in cultural humility through social interactions [44–47]. These social interactions provide learners with the experience to develop cultural awareness and humility as well as cross-cultural communication, allowing them to recognize and value local knowledge and advice over preconceptions as reflected in the results of this study [48–50]. Such experiences, as further described by Garcia et al. [47], can be foundational for the implementation of culturally sensitive care and effective patient-provider communication—both of which are integral to building trust and providing person-centred care [51]. Given the language barriers that often exist between local communities and GHCSL learners, the learners made it a priority to learn phrases in the local language as they entered clinical rotations, with many suggesting that this enabled deeper connections with local patient populations and better communication skills. Holmes et al. [42] has stated that working through cultural and language barriers is a necessary step towards fostering cultural humility and should not be avoided. While several studies have emphasized such assertions, it is crucial to consider the duration of the learner’s experience to avoid perpetuating misconception surrounding the knowledge, understanding, and degree of language skills learners from high-income countries are capable of developing during STEGH [8]. Pre-departure training, as implemented by the GHCSL program, can help in avoiding such misconception. As evident by Melby et al. [8], training sessions with local stakeholders are an essential component to the development of skills building in cross-cultural effectiveness and cultural humility, pertinent to the local context of the learners’ placement site.

Guided reflective exercise encouraged learners to compare and contrast the healthcare systems of their home country of Canada and their placement site. Our results reflect a complex understanding of the experiences and challenges of accessing oral health services among the local patient populations. Learners reflected on the adverse impact resource constraints had on the dissemination of care and the need to implement creative innovations to circumvent these challenges. Similarities and differences pertaining to the socioeconomic barriers of the patient populations, particularly poverty, and its connection to oral health inequities were emphasized and highlighted. Rivkin-Fish’s [9] critical analysis of service-learning suggested that learners who encounter “differences” are prompted to ask questions that shift from *how can we help?* to *why are conditions this way?* By delving into a more complex understanding of the local healthcare system, learners have the opportunity to gain insight into systemically produced inequities and the factors that play into these processes [9].

The GHCSL program gave opportunity to the learners to reflect on the prevalence of local endemic conditions such as HIV infection, along with the experience of HIV-related stigma. Evidence suggests that people living with HIV have difficulty access oral health services primary due to HIV-related stigma and discrimination [52–56]. Strong didactic training regarding the geographical context of local conditions must be included in pre-departure training to ensure culturally sensitive care [7]. This should be done with caution to ensure that learners avoid the development of preconceived notions and internalized stigma about the local patient population [7].

Experiences pertinent to global oral health disparities garnered numerous affirmations by the learners, with many reflecting on their motivation and dedication to engage in future service-learning and outreach initiatives after graduation. Previous studies have revealed that CSL—whether in a local or global context—exemplifies an approach that improves learners’ awareness to community oral health needs, reinstating the social contract of providing the best possible care to their patients. This could be due to their understating of the underlying complex socio-geographical determinates of oral health [41, 57–59]. However, caution is warranted to ensure that learners from high-income countries think less about ‘saving the world’ during their GHCSL placement and instead focus on the needs of the community and the risk-factors that drive these disparities [7].

Personal and professional growth was largely emphasized by the learners in their reflective essays. Consensus was made throughout the literature regarding the importance of experiential community service learning for learners’ professional growth as it provided them an opportunity to gain a better understanding of barriers to care and follow a patient-centred care model [60]. Such insight is suggested to be linked with personal growth, encouraging the development of future oral health providers who have the interpersonal skills necessary to provide individualized, high-standard care to patients [41].

The themes uncovered in the learners’ reflective essays were largely interconnected and did not appear to stand in isolation. Although for a brief period of time, engaging with diverse communities and patient needs, and witnessing socioeconomical risk factors in low-resource settings presented learners with the desire to understand and immerse in reflection related to global oral health disparities. The learner’s heightened social awareness and understanding of the local healthcare systems encouraged a deeper understanding of the unmet oral healthcare needs in low-resource communities in global settings.

While the results of this study are positive, the STEGH are not without limitations, especially when guideline principles, that focus on community benefit, are absent or present clear gaps [8]. The key ethical areas in STEGH development and implementation to optimize benefits for local communities should include: (1) emphasis on cross-cultural effectiveness skills and cultural humility, (2) bidirectional participatory relationships, (3) local capacity building, and (4) long-term sustainability [8]. The current GHCSL program is actively working towards the integration of each principle. To avoid the harmful impact of STEGH, a strong didactic component with robust pre-departure training was developed. This was done with the active engagement of local community stakeholders from the Makerere University and University of Rwanda in the development of program's goals and objectives. A rigorous learner selection process was integrated to ensure the selection of the most qualified candidates. The selection process not only emphasized excellent clinical and academic skills but also considered the motivation to serve, the ability to represent the school well, and the degree of cultural sensitivity as important parameters [7].

Instead of opting for a ‘one and done’ approach, learners from Canada were paired with local learners for ongoing projects to ensure sustainability and continuity of care. Clear expectations and guidelines pertinent to serving the global community were communicated, focusing on the community's needs rather than self-advancement [7]. However, given that the GHCSL program was piloted in the academic year of 2022–23, it is actively in the processes of promoting local capacity building, developing bidirectional relationships with the placement sites in Canada and learners from the Global South, and embedding itself within local community-led community project’s [8]. Post-placement debrief sessions, reflective narrative exercises, and formal program evaluation and feedback have been implemented by the Schulich School of Dentistry and Western University to ensure its optimal effectiveness and global impact.

### Limitations

There were limitations in this study. The first is the themes represent the preliminary findings of this pilot program and should be interpreted as such. Nevertheless, the study captured detailed experiences of the undergraduate dental learner’s participation in the GHCSL placements in East Africa, a focus not extensively researched. The second is the lack of inclusion of items prompted in the “Conceptualize and Critique” section of the program’s reflective assignment component, which may have promoted bias. There may be several reasons for this bias: learners may have been reluctant to presumptively represent the personal experiences of others in their personal writing, learners may not have gleaned this information because patients did not share it with them, or perhaps learners failed to recall the information when developing their reflections. As such, qualitative methodology to assess this section or the implementation of secondary analysis is warranted. The third is the program’s reflective assignment invites learners to make presumptions about the experiences of the local patient community—an evaluation that only the person with the lived experience can make. Although a key component of the reflection assignment was to document the observations they made regarding the experiences of the local patient population, it is essential that this limitation be acknowledged and considered. The last is that learners may have not necessarily expressed their genuine thoughts when developing their reflections, and rather reflected on what they perceived the GHCSL program director would want to read.

## Conclusion

Our results confirmed that the pilot GHCSL program played a positive role in the advancement of education and professional and personal development among undergraduate dentistry learners. Through the GHCSL experience, learners developed increased empathy, effective communication skills, and social awareness as well as a heightened desire to address global oral healthcare disparities. Providing learners with experiential learning experiences with guided reflection exercises can be a strong pedagogical tool in an undergraduate dental curriculum; however, further consideration on longitudinal engagement is necessary.

## Data Availability

The datasets used and/or analyzed during the current study available from the corresponding author on reasonable request.

## Appendix 1

### GHCSL application

GHCSL APPLICATIONS—2024

Adjudicator Name: _________________________________.

Application # _____________

**Table:**

Letter of Motivation (0–5)	Schulich / Western Representation (0–5)	Planned Learning Objectives (0–5)	Total (out of 15)	Resume: Concerns? Y/N	Transcripts: Concerns? Y/N	Clinical & Academic Reports: Concerns? Y/N	References: Concerns? Y/N

Elective Type:

Area of study (please note three choices).

Partner Site:

Have you travelled internationally for an exchange or outreach in the past?

If so, where and in what capacity?

Please write a letter of motivation as to why you are interested in this Program and why you would like to participate (max. 400 words):

Please outline how you plan to be a good representative for Schulich and Western during your time away.

Please identify at least three learning objectives you have for your time during the ICSL program. Please also explain how these personalized learning objectives relate to your current studies and career/academic goals.

GHCSL Reference Form Template

Referee Name:

Student Name:

Capacity referee knows student: Aimable was support staff in the hospital in Kenya while Arad was there for a Global Health Opportunity (June 2023).

1. Can you please speak to the student’s specific interest in learning about dentistry in a cross-cultural setting, from their conversations with you? What is their motivation to participate?
2. Can you provide examples of the student's approach to handling challenges in a professional setting, especially those related to teamwork and communication? How do you think this approach would work within a different culture?
3. Based on your knowledge, how has the student demonstrated a commitment to community service, particularly in the context of healthcare or dentistry?
4. Are there any concerns that we should be aware of?

## Appendix 2

### Assignment instructions

For your GHCSL placement, you are required to write a personal reflection to describe your personal experience through a ‘story-telling and critical incidence-based narrative’.

Here are the important points to consider:

Content and Context:

- Please describe comprehensive background information about allocated global health community placement e.g., their location, services, population served, background of service users, etc.

Conceptualize and Critique:

- Please reflect on your experiences through a *story telling and critical incidence-based narrative* meaning reflecting on individualized and personal *stories* of the community members your screened on site.
- Please do not share any personal details (e.g., name, date of birth, etc.). Share the 'story/stories' of their lived experiences. Guiding questions: what were their social/immigration backgrounds?; during your interaction with them, what were their perceived general and oral health challenges (e.g., transportation, childcare, etc.)?; what were their individualized risk factors (e.g., oral hygiene practices, diet, etc.), environmental factors (e.g., dental insurance, refugee status, mental health etc.), and systemic risk factors (e.g., racism, discrimination, anxiety, self-harm, homophobia, etc)?; what particular critical life incidence (e.g., migration, etc.) had an impact on their oral health/access to care?

Reflect and Present:

- Please reflect and present in detail about the challenges and social determinants of health and access to dental care within the global setting. How were these challenges similar or different to Canada? What were the similarities and differences with your local CSL program in Ontario?
- Please reflect on your understanding on how these experiences will impact you as future oral health provider.

You can use one or multiple stories to reflect on your experiences.

Please have a *separate section* on how this GHCSL experience supported your learning and personal growth.

This will be a *pass/fail* exercise with the following assessment criteria:

**I**. Background **Context**

o Provides sufficient local/global context that reader can get some sense of the personalized story/ies you intend to share.
o Clearly identifies themes of the person’s story, life or work experiences and its impact of oral health in the context of global health.
o Comprehensively describes person’s experiences and thoughts as per *your* understanding and interpretation in the context of global health.

**II**. ** Innovation and Interpretation.**

o Provides critical insight into their experiences and challenges of accessing oral health services in the context of global health.
o Conveys the importance of culturally sensitive, individualized, and person-centred care in the context for global health. Highlights the impact social and environmental determinants have in shaping access to oral/health care in Global South
o Provides critical and evidence-based interpretation of important themes associated with access to oral health in the context of global health
o Provides personal analysis of emerging themes and experiences pertinent to access to oral health in the context of global health.

**III**. **Application and Presentation.**

o Thoughtful and critical application of course readings and discussions to your witnessed stories and experiences.
o Excellent sentence construction and choice of words, correct spelling, good transition between paragraphs, and correct use of technical terms with concise writing and expression.
p Supported by peer-reveiwed citations (APA Style)

Instructions:

- Minimum one journal per week. If each journal should be at least 2 pages with times new roman 11pts, single space. If you were placed for 2 weeks, then you are required to submit 2 journals.
- *Word count:* 1500—2000 words
- *Format:* Single-spaced, times 12pts.
- Kindly submit this assignment to OWL by (*DATE)*

## Abbreviations

CSL: Community Service-Learning
GHCSL: Global Health Community Service-Learning
D3: Year 3
D4: Year 4
HIV: Human immunodeficiency virus
2SLGBTQ +: Two-Spirit, lesbian, gay, bisexual, transgender, queer (or questioning), and other sexual orientations and gender identities
STEGH: Short-term experiences in global health
